# Quantification of 17 Endogenous and Exogenous Steroidal Hormones in Equine and Bovine Blood for Doping Control with UHPLC-MS/MS

**DOI:** 10.3390/ph14050393

**Published:** 2021-04-21

**Authors:** Giovanni Caprioli, Michele Genangeli, Ahmed M. Mustafa, Riccardo Petrelli, Massimo Ricciutelli, Gianni Sagratini, Stefano Sartori, Fulvio Laus, Sauro Vittori, Manuela Cortese

**Affiliations:** 1 School of Pharmacy, University of Camerino, 62032 Camerino, Italy; giovanni.caprioli@unicam.it (G.C.); michele.genangeli@unicam.it (M.G.); ahmed.mustafa@unicam.it (A.M.M.); riccardo.petrelli@unicam.it (R.P.); massimo.ricciutelli@unicam.it (M.R.); gianni.sagratini@unicam.it (G.S.); sauro.vittori@unicam.it (S.V.); 2 Department of Pharmacognosy, Faculty of Pharmacy, Zagazig University, Zagazig 44519, Egypt; 3 Eureka Lab Division, 60033 Chiaravalle, Italy; sartori@eurekaone.com; 4 School of Bioscience and Veterinary Medicine, University of Camerino, 62032 Camerino, Italy; fulvio.laus@unicam.it

**Keywords:** HPLC-MS/MS, steroidal hormones, anti-doping, bovine blood, equine blood

## Abstract

A simple and fast analytical method able to simultaneously identify and quantify 17 endogenous and exogenous steroidal hormones was developed in bovine and equine blood using UHPLC-MS/MS. A total amount of 500 µL of sample was deproteinized with 500 µL of a mixture of methanol and zinc sulfate and evaporated. The mixture was reconstituted with 50 µL of a solution of 25% methanol and injected in the UHPLC-MS/MS triple quadrupole. The correlation coefficients of the calibration curves of the analyzed compounds were in the range of 0.9932–0.9999, and the limits of detection and quantification were in the range of 0.023–1.833 and 0.069–5.5 ppb, respectively. The developed method showed a high sensitivity and qualitative aspects allowing the detection and quantification of all steroids in equine and bovine blood. Moreover, the detection limit of testosterone (50 ppt) is half of the threshold admitted in plasma (100 ppt). Once validated, the method was used to quantify 17 steroid hormones in both bovine and equine blood samples. The primary endogenous compounds detected were corticosterone (range 0.28–0.60 ppb) and cortisol (range 0.44–10.00 ppb), followed by androstenedione, testosterone and 11-deoxycortisol.

## 1. Introduction

This paper is a follow-up study of a method developed by Genangeli et al. for the simultaneous determination of steroids in horse serum [1]. Endogenous and exogenous steroids are abused in animal-related sports, and they have a major role in regulating a wide number of endogenous signals in the organism [2,3]. Anabolic steroids are synthetic compound derivatives from testosterone. The primary function of anabolic steroids can be summarized into reproductive and sexual differentiation, homeostasis, growth, development, and regulation of metabolism and nutrient supply [3,4,5]. Doping control in horse racing and animal-related events poses different challenges, in comparison with other sports where humans are involved, because both performance-enhancing and performance-impairing substances (or methods) can be used to manipulate and change the outcome of the competition while the controls are not standardized and rarely applied [6]. This may be more predominant in an animal competition where the bets reach high values leading to an abuse of illegal substances in order to ensure the winning [6]. Nowadays, competitions or events involving animals like cattle or horses are increasing in popularity. As previously reported from Genangeli et al. [1], only eleven compounds are present in the list of prohibited substances with international thresholds in both urine or plasma [7]. Apart from substances like theobromine, dimethyl sulfoxide or salicylic acid, testosterone is still the only steroid regulated in plasma, and its threshold in plasma horses is 100 ppt quantitated as free testosterone [7]. The performance improvement or health conditions camouflage in horses, or other animals, are common techniques used before an animal trading or during a race. These substances can cause severe harm to the animal. As reported from Kavitha et al., the following are the adverse effects of anabolic steroids by topic: cardiovascular, endocrine and metabolic, gastrointestinal, genitourinary, hematologic and oncologic, neuromuscular and skeletal, neuropsychiatric, dermatologic and renal [1,8]. Qualitative evaluation of steroidal hormones and their metabolites and quantitation of these molecules is crucial for the correct diagnosis and/or treatment of several diseases and conditions, such as disorders of puberty, amenorrhea, polycystic ovary syndrome, infertility, osteoporosis, adrenal insufficiency, hypogonadism, cognitive dysfunction, cardiovascular diseases and hormone-related malignancies [9]. At the moment, current analytical procedures regarding the matter are self-developed analysis, expensive, complicated or long and time-consuming to be replicated in clinical laboratories. Additionally, these methods are often based on immunohistochemical analysis, with reduced sensitivity and the high possibility of false positive responses or wrong quantitation [10,11,12,13]. Genye et al. developed a method to detect and analyze 13 steroids in human urine using a quadrupole-Orbitrap LC-MS/MS [14]. Tajudheen et al. studied the separation of two anabolic substances using a reversed phase chiral chromatography approach [15]. Brian et al. published an article regarding novel liquid chromatography-tandem mass spectrometry methods for measuring steroids [16]. Youwen et al. focused on the separation of 16 testosterone and nandrolone esters in equine plasma [17]. Colton et al. developed a method for fast screening of anabolic steroids in horse urine [18]. From literature, it appears that the majority of methods for endogenous and exogenous steroid analysis is mainly focused on human samples [19]. Additionally, these methods are developed using either high-performance liquid chromatography-tandem mass spectrometry (HPLC-MS/MS) or gas chromatography (GC-MS) where HPLC-MS/MS is the perfect technique due to its extreme specificity and high sensitivity [20,21,22,23,24,25]. The majority of the procedures existing in literature are oriented towards horses or horse racing; here, the importance of developing a general method able to precisely quantify and qualify several endogenous and exogenous steroids in blood from different animals, sensitive and reproducible, with a short analytical time that can provide reliable results. Thus, our work aimed to set up a new UHPLC-tandem mass- based method to detect and quantify seventeen hormones and metabolites in equine and bovine blood. Detectable and quantifiable compounds included in the proposed method are as follows: androsterone (AND), androstenedione (ANDD), dehydroepiandrosterone (DHEA), testosterone (TEST), cortisol (COR), corticosterone (CoCo), aldosterone (ALDO), 11-deoxycortisol (11-DOC), 11-deoxycorticosterone (11-DCC), dihydrotestosterone (DHT), nandrolone (NAN), boldenone (BOL), stanozolol (STA), dexamethasone sodium phosphate (Desa NaP), dexamethasone isonicotinate, (Desa-Iso), methylprednisolone (MePre) and pregnenolone (PRE). One deuterated hormone (testosterone-D3) was used as an internal standard in order to make the analytical method more robust. All the compounds included in this methodology are different from the molecules included in analytical procedures reported in the literature but currently adopted for doping purposes [22,26]. The proposed procedure is not time-consuming with clear and simple sample preparation. Additionally, the method resulted in being sensitive, accurate and robust after a full validation. Hence, it could bring faster and cheaper analysis easily applicable from any external laboratory. The proposed procedure was fully validated and applied to the analysis of blood samples from different kinds of animals (mares, stallions, geldings and cows).

## 2. Results and Discussion

### 2.1. Setup of the Chromatographic and Mass Analyzer Conditions

After testing different chromatographic conditions, the best results were obtained with a solution of water and 0.1% of formic (mobile phase A) and acetonitrile and 0.1% of formic acid (mobile phase B). The use of other solvents as mobile phase B led to a worse separation among peaks, and the presence of formic acid in the mobile phase enhanced the ionization of the analytes in the ESI source, resulting in a greater sensitivity of the overall method. Due to the different chemical structure of the analytes, several chromatographic-gradient conditions were tested. The complete baseline separation of all peaks in the shortest time was achieved by using the chromatographic condition listed in Table 1, and described in the section ‘Liquid Chromatography-Tandem Mass Spectrometry’. A final column conditioning was also found to be essential for reproducibility of the retention time of the monitored compounds. A flow rate at 0.6 mL min^−1^ was the best option to achieve a good chromatographic separation in a short period of time. Different flow rates led to either a longer analytical time or an overlapping of peaks. As for the optimization of the chromatographic conditions, the mobile phases were chosen according to their influence in the ionization process occurring in the ESI source. The choice of acetonitrile and water with formic acid led to a significantly higher signal and ionization for all the compounds but also a good chromatography separation and resolution of peaks [27,28]. Additionally, papers in literature confirm that the addition of formic acid in a positive mode increases the response of target compounds [28,29]. In our case, we had an improvement of both ionization and chromatographic separation/resolution of peaks. The precursor and daughter ions obtained by injecting a standard solution of each compound are comparable with other methods found in the literature [16,18,22,26,30].

### 2.2. Method Validation

The proposed method was fully validated in terms of its analytical characteristics such as linearity, accuracy and precision, evaluation of the limit of detection (LOD) and limit of quantification (LOQ). Additionally, recovery and matrix were also investigated. The assessment of all these parameters is essential for the future application of the proposed method. All the concentrations were developed starting from the LOQ of every compound (Table 2).

### 2.3. Evaluation of the Stability of Steroids in Glass and Plastic

Several endogenous and exogenous steroids were tested for stability in glass and plastic. A standard concentration of 200 ppb of the compounds listed in Table 3 was prepared, and an aliquot of the before mentioned mix was transferred into four plastic vials and four glass vials and stored at −4 °C. The first vial was immediately analyzed and the other four were analyzed over the following 3 days. As reported in Table 3, immediately after one day, the concentration of the steroids stored in the glass test tube dropped with a loss of >98%, suggesting an interaction of the analytes with the glass of the container.

### 2.4. Precision and Linearity

Precision is known to be the closeness of agreement between independent test results obtained under stipulated conditions [1,31]. It is usually reported regarding standard deviation (SD) or relative standard deviation (RSD) [1,31]. The precision (intra-day and inter-day) was calculated from data obtained during a three-day validation (Table 4) of five daily repetitions using four concentrations from the LOQ to the U1 (LOQ, CM, CU and U1). The outcome is expressed according to the coefficient of variation (CV%). The CV resulted to be included in the range of 0.48–18.78% (Table 4). The inter-day precision (*n* = 5) expressed in relative standard deviation percent (RSD) was also satisfactory. The LOQ displayed RSD in the range of 10.86–18.37%, the CM resulted in an RSD of 2.62–18.78%, the CU showed an RSD of 3.52–18.40% and U1 had an RSD within 0.48–9.41% (Table 4). To calculate the linearity of the proposed method, two calibration curves were created using all the concentrations between LOQ and CU (low-range standard curve, 5 points, 5-day validation) and all the concentrations between the LOQ and U1 (high-range standard curve, 6 points, 5-day validation). The high-range curve was used to test the linearity in a more extense dynamic range. The linearity is expressed as the coefficient of linear regression (*R*^2^), and it is higher than 0.99% (Table 4).

The LODs and LOQs for all the compounds included in this analytical procedure displayed values in the range of 0.023–1.833 and 0.069–5.5 ppb, respectively. These values are similar and sometimes lower when compared with a limit of detection and quantification reported in the literature [22,26,32]. Additionally, the steroids and metabolites included in this procedure were chosen due to their frequency of usage and because they were partially included in procedure already present in the literature. Moreover, LOQ for testosterone is equal to 0.05 ppb, twice lower concerning 0.1 ppb (or 100 ppt), which is the threshold for plasma samples of young horses (geldings) [7].

### 2.5. Accuracy

Accuracy is known to be the closeness of agreement between a test result and the accepted reference value of the property being measured [1,33].

The intra and inter-day accuracy were calculated from the C1, C2 and U1 concentrations, from the data obtained during a three-day validation. The results are listed in Table S1 and expressed in terms of ‘relative error percentage’ (RE%). The RE% for all the analytes were within the range, 0.92–13.90% (Table S1). The inter-day (*n* = 5) accuracy was also satisfactory. Precisely, at the C1 concentration, the RE% values were in the range 6.08–13.64%; at the C2 concentration, the RE% values were 1.23–7.12%, and at the U1 concentration, the RE% values were 1.10–4.19%.

### 2.6. Recovery

Recovery was studied by spiking clean equine and bovine blood with a mixture standard of the 17 hormones. The recovery value was obtained using the following formula: ((*A*_se_ − *As*_blank_)/*A*_std_) × 100, where *A*_se_ is the area about the serum enriched with a low concentration (C1 and CM) of all the compounds, *A*_blank_ is the area of analytes detected in the serum and *A*_std_ is the area of a mixture standard of all the compounds dissolved in methanol. The recoveries obtained by spiking the matrix at the CM concentration were in the range of 86.75–98.32%, with a CV lower than 5.04% (Table S2). Moreover, the recoveries at a concentration of C1 were in the range 85.60–99.39%, with a CV lower than 8.21% (Table S2).

### 2.7. Matrix Effect

**Figure 1:**
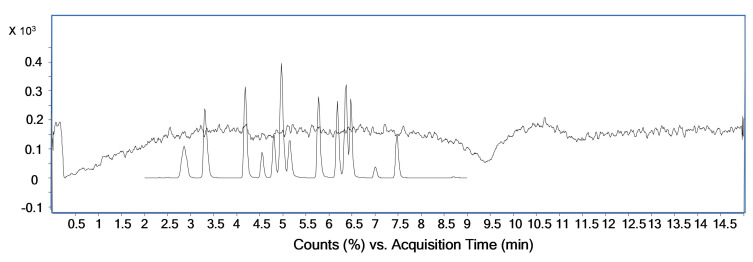
Post-column infusion of steroids serum-free and a mixture standard of all searched compounds in high-performance liquid chromatography-grade methanol.

Matrix is often responsible for a reduced or an increased signal/ionization (ion suppression/enhancement) in mass spectrometry [34]. These effects can strongly affect and compromise the quality and reproducibility of biological samples when injected and studied with LC-ESI-MS. To test the matrix effect, we performed a test known as “post-column infusion”. This test, according to the literature, is one of the best techniques used to obtain qualitative information about matrix effects [35]. A methanol mixture of all the compounds at the medium concentration (CM) was injected in the ESI source using a micropump. Simultaneously, an injection of purified and deproteinized blood was performed. As shown in Figure 1, the signal is constant for almost all the chromatographic time, with the exception for a signal suppression at 9.5 min. All the compounds have a retention time shorter than 9.5 min; hence, the matrix does not cause ion suppression or enhancement effects in our method.

### 2.8. Specificity

In order to quantify the specificity of the proposed method, we controlled the retention time of parent/daughter ions for all the analytes over time. For each compound, we examined the chromatographic retention time regarding reproducibility, for a number of five times over a five-day period (*n* = 25). The RSD regarding the retention time was stable with an average percent value ≤ 0.97%. Specific parent/daughter ion transitions were identified for each steroid, and the MRM transitions with the most abundant product ion were selected for quantitation, and the other product ion was selected for qualification (Table 1). High specificity was achieved.

### 2.9. Application and Testing of the Developed Method to Equine and Bovine Blood

The method described in this paper was successfully applied to thirty-three samples provided from four control agencies located in the south of Italy (Groups A, B, C, D). The high sensitivity and the quantitative aspects of the proposed method allowed the detection of most of the compound in the equine and bovine blood samples. The analysis was performed in blind, and due to privacy, we do not know if samples were bovine or equine. Due to the blind analysis and the thresholds changing between gender, species and age of the animals, we could not compare our results with international guidelines [36].

A total of thirty-three animals were analyzed in triplicate; percent RSDs in all cases were lower than 13.66%. Moreover, the mean values of the analytes found in the various samples are reported in Table 5. Only androsterone and DHEA were not detected in any samples. The main compounds found in the four groups of samples were cortisol (range 0.44–10.00 ppb), followed by corticosterone, androstenedione and dexamethasone isonicotinate. Overall, the level of exogenous steroids is below the thresholds from different guidelines and papers in the literature (pregnenolone, stanozolol and nandrolone < 1 ppb, boldenone < 15 ppb, testosterone < 20 ppb for geldings in plasma and <55 ppb for mares and fillies not in foal [6,37,38]). None of the samples showed levels of exogenous substances above 1 ppb, with the exception of ME-PRE in group C. Some animals showed traces of some of the exogenous steroids. In particular, DESA-NA-P is present only in the group A. PRE is present only in two samples belonging to group A. BOL was present only in three samples and not in group C. NAN was present in three samples belonging to group A and D. Our findings are the first step for a lager monitoring project on the presence of these exogenous compounds in mammals and their healthy effects.

## 3. Materials and Methods

### 3.1. Disposable Chemicals and Materials

All the detected compounds used in this paper, including the internal standard, were ordered from Sigma-Aldrich (Milano, Italy) with a purity > 99%. An individual stock solution of each compound was prepared by the dissolution of 0.5 mg of every single molecule in 0.5 mL of HPLC-grade methanol (Carlo Erba, Milano, Italy). Other solutions were obtained from the dilution of the stock solutions in methanol. HPLC-grade acetonitrile was purchased from Carlo Erba (Milano, Italy). HPLC-grade formic acid (99%) was obtained from Merck (Darmstadt, Germany). Deionized water (>18 MΩ cm resistivity) was obtained by purification of water with a Milli-Q SP system (Millipore, Bedford, MA, USA). Sterile test tubes were purchased from Becton–Dickinson (Franklin Lakes, NJ, USA). The analytical procedures were carried out in polypropylene vials, test tubes and plastic centrifuge tubes in order to preserve the concentration and stability of the hormones. The glass has demonstrated that it could interfere with those molecules as reported in the section “*Evaluation of the stability of steroids in glass and plastic*” (Results and Discussion, Table 3).

### 3.2. Collection of Equine Blood

The equine and bovine blood used for the method optimization was collected from healthy horses and cows from the Unicam veterinary hospital in Matelica (MC) and stored in vials with EDTA or Lithium heparin. The blood was refined using activated charcoal to obtain a standard hormone-free-matrix and stored at −4 °C. The tested blood was obtained from several veterinary hospitals from the south of Italy, stored at −4 °C if analyzed within two days or stored at −20 °C if analyzed after two days.

### 3.3. Preparation of Steroids-Free Blood and Sample Preparation

The blood (50 mL) was kept under magnetic agitation overnight, with 1 g of charcoal to create steroids-free blood used in the development and validation steps, as reported by Genangeli et al. [1,39]. The solution was then let settle for 10 min, and the clear blood without visual residues of charcoal was transferred into a clean 50 mL plastic test tube. To obtain clean blood from the charcoal residues, this last step was repeated for additional time or until the no residues of charcoal were present on the bottom of the test tube. The hormone-free blood was stored at −20 °C.

A total of 500 μL of blood was denaturated using 500 µL of a denaturing solution made of methanol (MeOH) and 5 g/L of zinc sulfate (ZnSO_4_) and internal standard (testosterone d3, 1000 ppb) in Eppendorf tubes. The deproteinizing solution was prepared leaving the methanol and zinc sulfate under magnetic agitation overnight and then filtering the solution with a paper filter. The solution was then agitated with vortex for approximately 1 min to avoid the formation of blood clotting. The solution was centrifuged for 15 min at 13,000 rpm, and then the liquid was transferred into polypropylene test tubes. The obtained solution was evaporated under nitrogen gas flow, then rebuilt using 50 µL of 25% methanol and centrifuged again for 10 min at 13,000 rpm, and finally pipetted in high-recovery vials and injected in the UHPLC-ESI-MS/MS system.

### 3.4. UHPLC-ESI-MS/MS

The UHPLC system used was an Agilent 1290 infinity series coupled with an Agilent Technologies ESI-triple quadrupole 6420 (Santa Clara, CA, USA). The analytes were separated using a Zorbax RRHD C18 as an analytical column (50 × 2.10 mm, the internal diameter of 1.8 µm), also from Agilent Technologies (USA). The mobile phases adopted for the analysis are water (A) and acetonitrile (B) both containing 0.1% formic acid. The mobile phases were kept at a constant flow of 0.6 mL min^−1^ with a gradient elution of: 0 min 15% B, 2.5 min 25% B, 5 min 35% B, 7 min 50% B, 9 min 90% B and 11 min 15% B, and kept at 15% B until the end of the run (15 min). Five µL of samples were injected with an auto-sampler. The column was kept at 20 °C, and the drying gas in the ESI source 300 °C. The gas flow was 12 L min^−1^, the pressure of the nebulizer was 40 psi, and the capillary voltage was 4000 V (negative and positive). Detection was performed in the ‘multiple reaction monitoring’ (MRM) mode dividing the runtime into four segments as reported in Table 1. The most abundant daughter ion was used for quantification purposes, and the rest of the daughter ions were used for qualification purposes. All the information regarding the compounds, abbreviation and settings of the mass analyzer are reported in Table 1.

### 3.5. Method Validation Settings

For the method evaluation and validation, seven concentrations of each analyte were used, starting from the limit of detection (LOD), limit of quantification (LOQ), then a series of low, medium and high concentrations (C1, CM, C2, CU). All the concentrations listed before, except for LOD, were used in the low-range standard curve (5 points); and for the high-range standard curve, one additional ‘upper’ concentrations (U1) was included (total of 6 points). All the concentrations divided by the compounds are summarized in Table 2.

Several concentrations were tested in order to find the LOQ. Precisely, ten concentrations were tested, and the LOQ values are in a range from 5.5 ppb for the DHEA and 0.069 ppb for the DESA ISO (data not shown).

### 3.6. Internal Standards

One deuterated internal standard was introduced as a control to increase the robustness of the method. The standard was added prior to the deproteinization step at the concentration of 1000 ppb.

## 4. Conclusions

A new UHPLC-MS/MS method was developed, permitting the detection of 17 endogenous and exogenous anabolic substances in equine and bovine blood samples. Most of the compounds in the current method are different from those reported in the literature, especially the ones of exogenous origin, and largely adopted to modify the condition of the considered animals. The high sensitivity of the method permitted the detection of several exogenous steroids in real samples, and can be considered the premise of a larger monitoring activity in order to obtain a robust statistical confirmation of our data, and evaluate the healthy effects of these species in the founded concentration levels. In addition, from the analytical point of view, the sample preparation is time-saving, fast and intuitive. With the proposed analytical method, it is possible to simultaneously monitor, quantify and qualify a large number of steroids presenting various steroid substructures in a short time (15 min chromatographic run) from blood samples. Moreover, the validation process demonstrated excellent performance regarding specificity, sensitivity (LOQ in the range of 0.069–5.5 ppb) and linearity. For the first time, a stability study of steroids was performed, revealing an interaction between our target analytes and the glassy wall of the storage container, then, the use of plastic materials is necessary for this purpose. The method was extended to detect most of the steroid esters in two animal species: horses and cattle. The results demonstrated the ruggedness of the method with respect to the biological variability of samples. The main steroids found in the four types of samples were cortisol, followed by corticosterone, androstenedione and dexamethasone isonicotinate. Androsterone and DHEA were not detected in any sample. In conclusion, the present method allows identification and quantification of steroids and performance increasing hormones, and it could be used when fraudulent use is suspected in racing animals, in an equine trade or to control the healthy state of these animals, including cattle.

## Figures and Tables

**Table 1:** Ultra-high-performance liquid chromatography-tandem mass spectrometry acquisition parameters (multiple reaction monitoring mode) used for the analysis of steroidal hormones and metabolites.

Compound	Abbreviation	Time Window (Minute)	Precursor Ion ^a^ (*m*/*z*)	Product Ion (*m*/*z*)	Fragmentor (V)	Collision Energy (V)	Dwell Time (Milli- Second)
Dexamethasone Sodium Phosphate	Desa-NA-P	2.0–3.7	473.11	435.2 355.2	97	8	200
Cortisol	CORT	2.0–3.7	363.01	121.1 327.2	136	24	200
Aldosterone	ALDO	2.0–3.7	361.41	343.2 315.2	116	16	200
Pregnenolone	PRE	2.0–3.7	361.41	343.2 105.0	87	4	200
Methylprednisolone	ME-PRE	3.7–5.5	375.01	357.2 323.2	92	4	180
11-Deoxycortisol	11-DOC	3.7–5.5	347.51	109.10 97.2	141	32	180
Corticosterone	COCO	3.7–5.5	347.01	329.2 329.2	111	12	180
Stanozolol	STA	3.7–5.5	329.51	81.10 95.10	170	50	180
Boldenone	BOL	3.7–5.5	287.41	121.00 135.00	107	24	180
Nandrolone	NAN	3.7–5.5	275.10	109.10 82.90	100	28	180
Dexamethasone isonicotinate	DESA-ISO	5.5–6.8	498.61	47.20 124.0	121	8	200
11-Deoxycorticosterone	11-DCC	5.5–6.8	331.01	97.10 109.1	117	20	200
Dihydrotestosterone	DHT	6.8–9.0	273.10	255.30 147.0	159	15	200
Testosterone	TESTO	5.5–6.8	289.01	97.10 109.1	131	20	100
Androstenedione	ANDD	5.5–6.8	287.01	97.10 109.1	131	24	200
Dehydroepiandrosterone	DHEA	5.5–6.8	271.01	253.10 253.2	92	8	200
Androsterone	ANDRO	6.8–9.0	291.41	273.20 255.2	78	4	200
Testosterone–d3	d_3_-TESTO	5.5–6.8	292.00	97.00	135	25	120

^a^ For every compound, the first product ion was used for quantitation and the second for qualification.

**Table 2:** Values of the concentrations used for method validation for each analyte.

Compound	LOD	LOQ	C1	CM	C2	CU	U1
	ppb						
DESA-NA-P	0.333	1.00	10	50	100	500	1000
COR	0.183	0.55	11	55	110	550	1100
ALDO	0.183	0.55	11	55	110	550	1100
PRE	0.033	0.10	10	50	100	500	1000
ME-PRE	0.067	0.20	20	100	200	1000	2000
11-DOC	0.037	0.11	11	55	110	550	1100
COCO	0.167	0.50	10	50	100	500	1000
STA	0.033	0.10	10	50	100	500	1000
BOL	0.167	0.50	10	50	100	500	1000
NAN	0.333	1.00	10	50	100	500	1000
DESA-ISO	0.023	0.069	6.9	34.5	69	345	690
11-DCC	0.037	0.11	11	55	110	550	1100
TESTO	0.037	0.05	11	55	110	550	1100
ANDD	0.333	1.00	10	50	100	500	1000
DHEA	1.833	5.50	11	55	110	550	1100
ANDRO	0.733	2.20	22	110	220	1100	2200
DHT	1.833	5.50	11	55	110	550	1100

**Table 3:** Loss of compounds in plastic vs. glass containers.

	DAY 1		DAY 2	
	Loss in Plastic (%)	Loss in Glass (%)	Loss in Plastic (%)	Loss in Glass (%)
COR	<1	99.63	<2%	>99.90
ALDO	<1	94.29	<2%	>99.90
11-DOC	<1	99.94	<2%	>99.90
COCO	<1	99.76	<2%	>99.90
11-DCC	<1	99.88	<2%	>99.90
PRE	<1	99.97	<2%	>99.90
ANDRO	<1	99.52	<2%	>99.90
TESTO	<1	99.85	<2%	>99.90
ANDD	<1	99.83	<2%	>99.90
DHT	<1	99.12	<2%	>99.90
DHEA	<1	99.65	<2%	>99.90

**Table 4:** Intra-day and inter-day precision expressed in CV% and linearity expressed in R^2^.

Compound	Limit of Quantification		Medium Concentration		CU		U1		Linearity
	Intra-Day (CV%)	Inter-Day (CV%)	Intra-Day (CV%)	Inter-Day (CV%)	Intra-Day (CV%)	Inter-Day (CV%)	Intra-Day (CV%)	Inter-Day (CV%)	*R* ^2^
DESA-NA-P	10.86	14.16	6.74	7.58	2.62	4.12	1.02	1.48	0.99326
COR	16.47	18.49	9.50	10.35	6.49	8.24	3.56	4.49	0.99939
ALDO	14.45	17.52	6.95	8.40	6.79	7.63	6.07	6.85	0.99995
PRE	15.84	17.41	10.07	10.91	2.78	3.69	1.18	2.37	0.99922
ME-PRE	15.93	17.45	15.04	17.85	6.32	18.40	3.26	8.12	0.99912
11-DOC	17.10	17.87	6.63	7.72	6.93	7.53	5.48	6.73	0.99954
COCO	10.34	14.57	7.97	10.63	1.56	4.51	0.48	2.28	0.99991
STA	13.25	14.95	5.73	9.11	3.15	5.24	1.18	4.20	0.99941
BOL	15.83	17.89	11.85	16.66	3.71	18.05	2.03	6.97	0.99658
NAN	17.35	18.62	7.12	14.75	5.49	8.13	2.26	5.83	0.99861
DESA-ISO	17.43	18.37	9.77	18.99	7.88	9.65	4.49	6.41	0.99981
11-DCC	16.60	18.07	16.06	18.78	12.21	18.27	7.21	9.41	0.99953
TESTO	11.76	18.17	11.40	16.89	6.35	15.06	4.39	5.72	0.99841
ANDD	10.08	16.66	14.04	18.46	8.56	9.88	6.29	8.06	0.99987
DHEA	14.21	17.84	9.99	18.64	4.67	8.49	2.18	3.26	0.99970
ANDRO	16.81	17.77	5.34	8.23	4.30	4.80	2.93	3.21	0.99940
DHT	13.97	17.10	9.07	9.19	6.31	6.57	4.29	5.30	0.99997

**Table 5:** Content of endogenous and exogenous steroids in equine and bovine blood samples, expressed in ppb.

Exogenous Steroids								Endogenous Steroids									
Animal	DESA-NA-P	PRE	ME-PRE	STA	BOL	NAN	DESA-ISO	11-DCC	TESTO	ANDD	DHEA	ANDRO	DHT	COR	ALDO	11-DOC	COCO
	**ppb**																
**A 01**	0.152	-	0.470	0.395	-	0.237	0.245	-	0.145	0.481	-	-	-	4.207	1.038	0.190	0.319
**A 02**	0.134	-	0.478	0.397	-	-	0.243	-	0.142	0.482	-	-	-	0.668	1.095	-	0.330
**A 03**	-	0.444	0.558	0.392	-	-	0.242	0.183	0.138	0.497	-	-	-	3.631	-	0.179	0.288
**A 04**	-	-	-	0.419	-	-	0.247	-	-	0.478	-	-	-	6.578	0.846	0.201	0.587
**A 05**	0.130	-	-	0.393	-	-	0.246	-	-	0.484	-	-	-	2.363	-	-	-
**A 06**	0.100	-	-	0.390	-	-	0.242	0.178	-	0.461	-	-	-	3.247	-	0.180	-
**A 07**	-	-	-	-	-	-	0.241	0.187	-	0.497	-	-	-		-	-	-
**A 08**	-	-	-	-	-	-	-	0.194	0.142	0.436	-	-	-	1.670	-	-	-
**A 09**	0.071	-	-	-	-	-	-	-	-	0.479	-	-	-	0.444	-	-	0.334
**A 10**	-	-	-	0.462	-	-	0.250	0.202	0.146	0.500	-	-	-	0.465	-	-	0.351
**A 11**	-	-	-	-	-	-	0.241	-	0.137	0.532	-	-	-	2.082	-	-	0.315
**A 12**	0.094	-	-	-	-	-	0.243	-	-	0.571	-	-	-	0.435	-	-	0.393
**A 13**	0.170	-	-	0.393	-	-	0.244	-	-	0.561	-	-	-	1.956	-	-	0.375
**A 14**	0.121	-	-	0.387	-	-	0.247	-	-	0.443	-	-	-	3.935	-	-	0.491
**A 15**	0.196	0,472	-	-	0.064	0.291	0.258	0.229	-	0.515	-	-	-	3.486	-	0.197	0.383
**B 01**	-	-	0.87	-	0.02	-	-	0.39	-	-	-	-	0.98	5.96	-	0.53	0.49
**B 02**	-	-	-	-	-	-	-	0.39	0.23	0.60	-	-	1.87	1.29	0.70	-	0.40
**B 03**	-	-	-	-	-	-	0.47	0.39	0.22	-	-	-	1.95	0.99	-	-	0.44
**B 04**	-	-	0.83	-	-	-	-	-	0.22	-	-	-	1.06	5.62	-	-	0.41
**B 05**	-	-	0.95	-	-	-	0.47	-	0.21	-	-	-	3.26	1.67	-	-	-
**B 06**	-	-	0.86	-	-	-	0.47	-	-	-	-	-	-	1.35	-	-	-
**C 01**	-	-	1.33	-	-	-	0.39	-	0.31	0.62	-	-	-	10.00	0.79	0.48	0.60
**C 02**	-	-	1.05	-	-	-	0.40	-	-	0.64	-	-	-	3.61	-	0.45	0.54
**C 03**	-	-	1.07	0.27	-	-	0.40	-	-	-	-	-	-	1.17	-	-	-
**C 04**	-	-	1.08	-	-	-	-	-	0.31	-	-	-	-	2.36	-	-	0.54
**C 05**	-	-	1.08	-	-	-	0.40	-	-	-	-	-	-	0.88	-	-	0.45
**C 06**	-	-	1.02	-	-	-	0.40	-	-	0.62	-	-	-	4.96	-	0.46	0.58
**D 01**	-	-	0.876	-	0.024	0.531	-	0.397	-	-	-	-	0.986	5.967	-	0.531	0.497
**D 02**	-	-	-	-	-	-	-	0.396	0.236	0.604	-	-	1.872	1.296	0.702	-	0.401
**D 03**	-	-	-	-	-	-	0.3	0.394	0.221	-	-	-	0.959	0.992	-	-	0.448
**D 04**	-	-	0.834	-	-	-	-	-	0.226	-	-	-	1.060	5.62	-	-	0.418
**D 05**	-	-	0.953	-	-	-	-	-	0.219	-	-	-	-	1.671	-	-	-
**D 06**	-	-	0.866	-	-	-	0.4	-	0.213	-	-	-	-	1.356	-	-	-

## Data Availability

Not applicable.

## Supplementary Materials

The following are available online at https://www.mdpi.com/article/10.3390/ph14050393/s1, Table S1: Intra-day and inter-day accuracy expressed in RE%, Table S2: Percent recovery and reproducibility at two fortification levels.
