# Supplementary material for: Quantification of 17 Endogenous and Exogenous Steroidal Hormones in Equine and Bovine Blood for Doping Control with UHPLC-MS/MS
Source: Pharmaceuticals (Basel). 2021 Apr 21;14(5):393. doi: 10.3390/ph14050393 (PMC8143330; doi:10.3390/ph14050393)
Supplement: Supplementary file 1 [file pharmaceuticals-14-00393-s001.zip › pharmaceuticals-1178159-supplementary.pdf]

Supplemental file

Table 1S. Intra-day and inter-day accuracy expressed in RE%.

Compound	C1		C2		U1	
	Intra-day (RE%)	Inter-day (RE %)	Intra-day (RE %)	Inter-day (RE %)	Intra-day (RE %)	Inter-day (RE %)
DESA-NA-P	6.1	6.3	1.2	1.5	0.8	1.0
COR	10.5	11.1	3.7	4.1	1.4	3.0
ALDO	8.9	9.3	2.7	4.0	1.7	1.9
PRE	7.0	9.8	5.8	6.6	2.6	3.5
ME - PRE	8.2	9.0	2.4	4.9	1.3	2.4
11 - DOC	10.5	11.7	3.6	5.1	1.5	3.0
COCO	5.2	5.9	1.1	1.4	1.7	1.3
STA	12.6	13.5	5.3	5.5	3.2	3.3
BOL	7.9	9.2	5.6	5.9	2.4	3.1
NAN	13.0	13.2	3.7	4.3	1.2	2.3
DESA - ISO	10.8	10.4	5.7	7.1	3.6	4.2
11 - DCC	12.3	13.6	4.6	6.7	2.5	2.8
TESTO	8.6	13.4	5.0	5.9	2.9	3.9
ANDD	7.9	9.0	4.5	4.9	3.4	3.9
DHEA	10.3	10.8	2.3	3.9	1.4	2.9
ANDRO	9.0	10.3	3.2	3.6	1.1	2.0
DHT	6.4	8.5	3.0	4.6	1.3	2.4

Table 2S. Percent recovery and reproducibility at two fortification levels.

Compound	CM		C1	
	Recovery (n = 3)	CV%	Recovery (n = 3)	CV%
DESA-NA-P	97.7	0.9	91.1	3.1
COR	96.6	1.4	89.8	5.7
ALDO	95.1	0.9	92.1	3.6
PRE	93.1	0.7	90.2	2.5
ME - PRE	98.3	2.0	95.6	6.7
11 - DOC	97.6	1.5	93.3	4.3
COCO	92.8	5.0	86.8	7.0
STA	95.1	1.0	90.9	3.4
BOL	96.2	1.2	93.3	5.8
NAN	98.3	3.5	96.4	8.2
DESA - ISO	97.2	4.0	94.5	6.7
11 - DCC	95.9	3.1	92.8	4.7
TESTO	95.7	0.8	93.4	2.6
ANDD	95.7	1.1	93.3	4.0
DHEA	96.0	3.1	90.2	6.9
ANDRO	95.7	3.6	93.6	7.7
DHT	91.1	1.1	88.5	5.5
